# Supplementary material for: Correlation between Physical Performance and Tensiomyographic and Myotonometric Parameters in Older Adults
Source: Healthcare (Basel). 2023 Jul 31;11(15):2169. doi: 10.3390/healthcare11152169 (PMC10418601; doi:10.3390/healthcare11152169)

Supplementary Material S1

5XSST, five-times sit to stand test; Dm, maximal radial muscle displacement; RF, rectus femoris; St, stiffness; SPPB, short physical performance battery; VL, vastus lateralis.

Supplementary Material S1a: Correlation diagram SPPB and Dm-RF

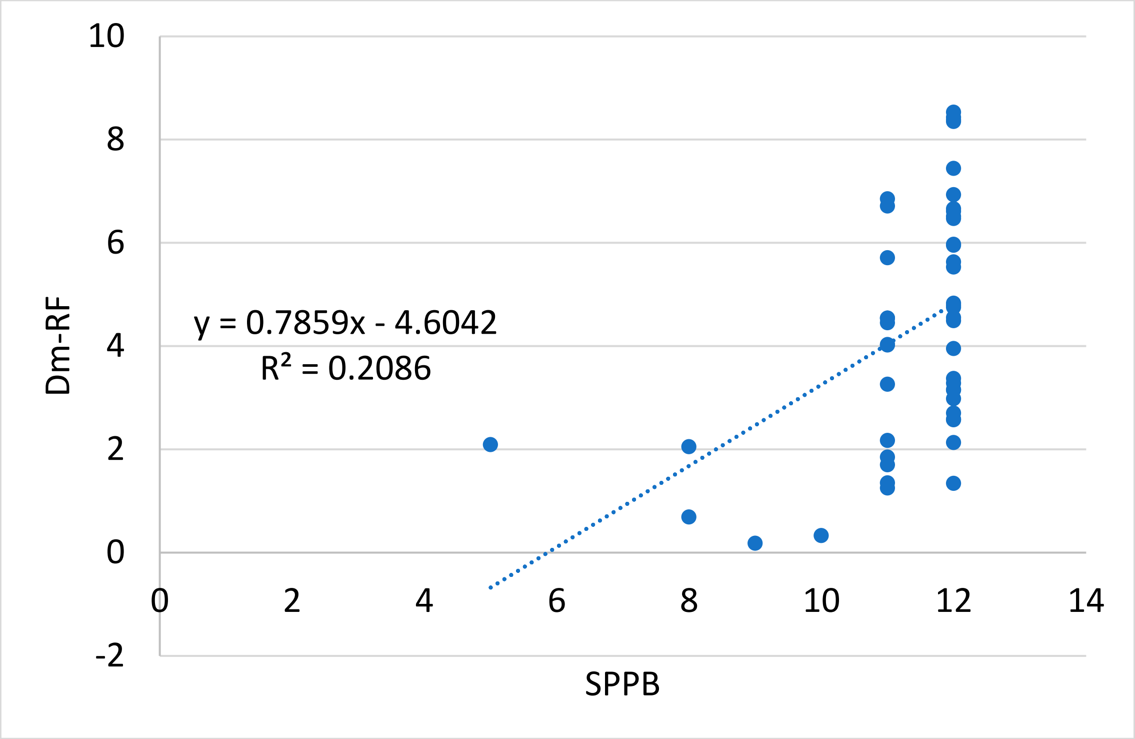

Supplementary Material S1b: Correlation diagram SPPB and Dm-VL

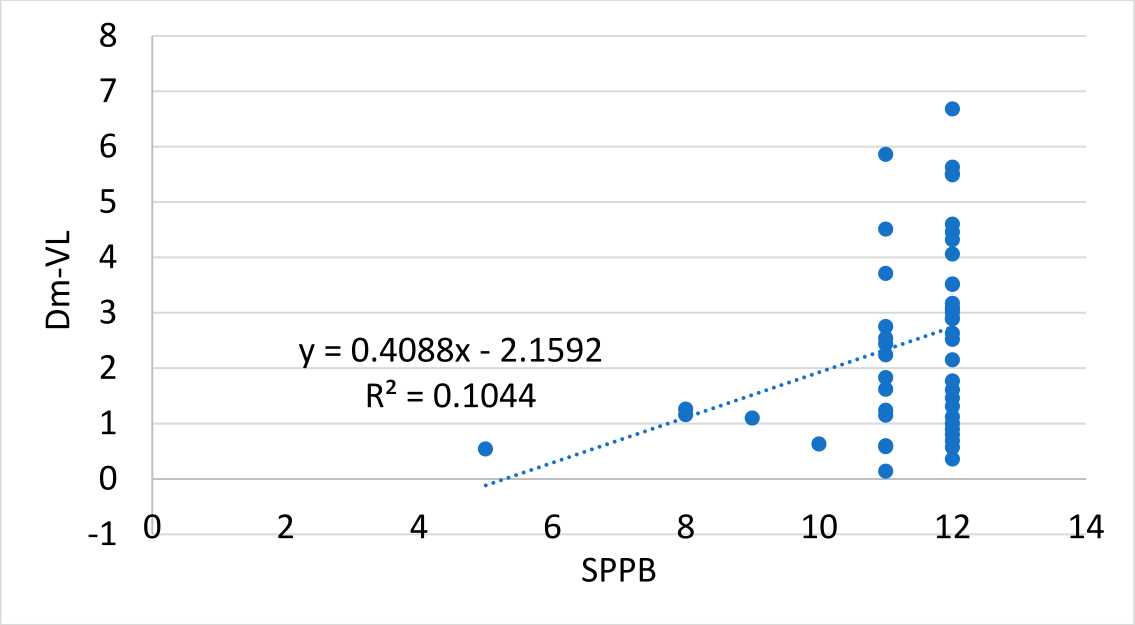

Supplementary Material S1c: Correlation diagram 5XSST and Dm-RF

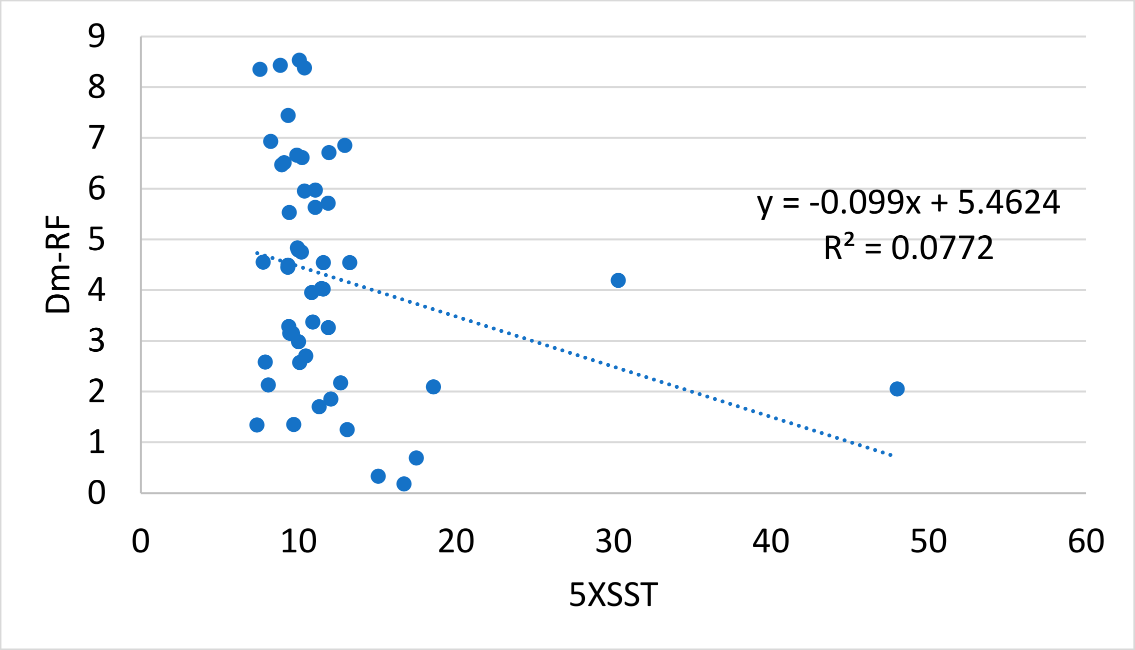

Supplementary Material S1d: Correlation diagram 5XSST and Dm-VL

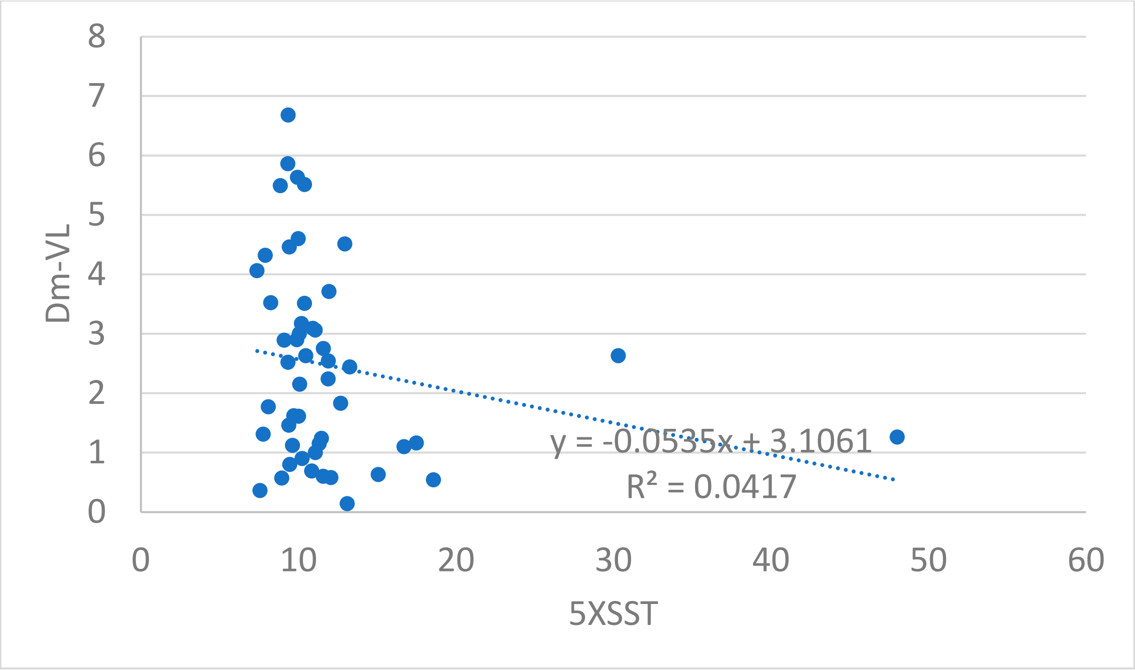

Supplementary Material S1e: Correlation diagram 5XSST and St-VL

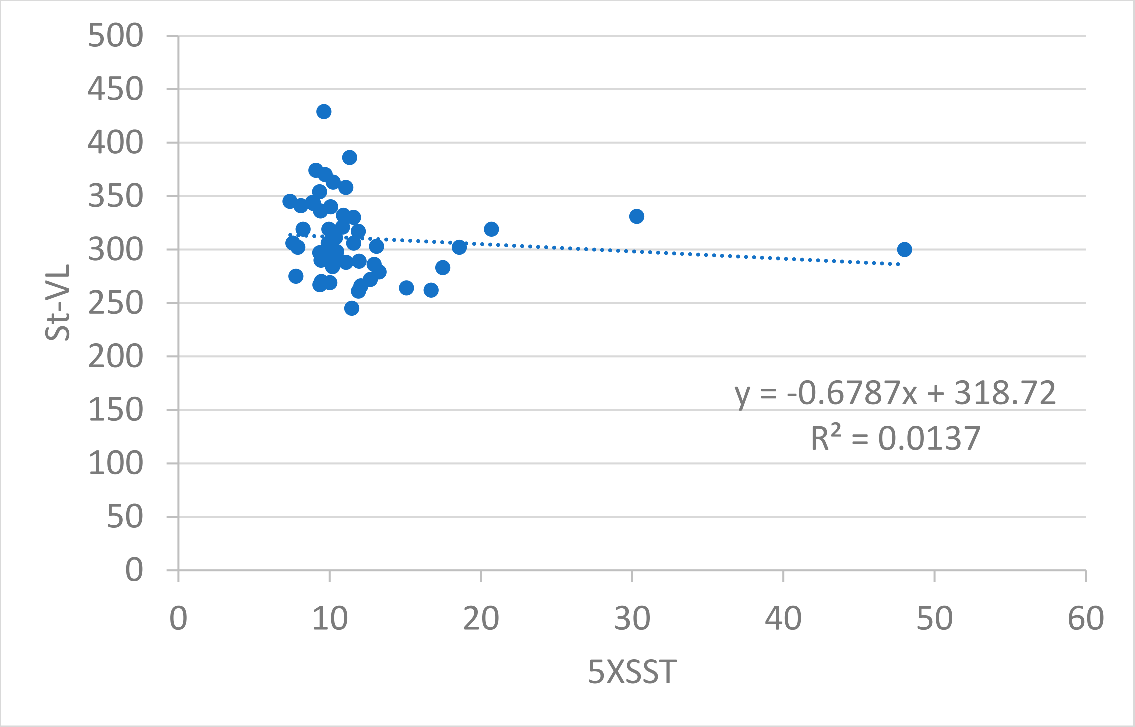

Supplement: Supplementary file 1 [file healthcare-11-02169-s001.zip › healthcare-2470218-supplementary.pdf]
